# TDP-43 aggregation inside micronuclei reveals a potential mechanism for protein inclusion formation in ALS

**DOI:** 10.1038/s41598-019-56483-y

**Published:** 2019-12-27

**Authors:** Cristian A. Droppelmann, Danae Campos-Melo, Alexander J. Moszczynski, Hind Amzil, Michael J. Strong

**Affiliations:** 1 Molecular Medicine Group, Robarts Research Institute, Schulich School of Medicine & Dentistry, Western University, London, Ontario Canada; 2 Department of Clinical Neurological Sciences, Schulich School of Medicine & Dentistry, Western University, London, Ontario Canada

**Keywords:** Amyotrophic lateral sclerosis, Mechanisms of disease

## Abstract

Amyotrophic lateral sclerosis (ALS) is a devastating progressive neurodegenerative disease with no known etiology. The formation of pathological protein inclusions, including RNA-binding proteins such as TDP-43 and rho guanine nucleotide exchange factor (RGNEF) are a hallmark of ALS. Despite intensive research, the mechanisms behind protein aggregate formation in ALS remains unclear. We have investigated the role of metabolic stress in protein aggregate formation analyzing how it is relevant to the co-aggregation observed between RGNEF and TDP-43 in motor neurons of ALS patients. Metabolic stress was able to induce formation of micronuclei, small nuclear fragments, in cultured cells. Notably, we observed the formation TDP-43 protein inclusions within micronuclei that co-aggregate with RGNEF and can be released to the cytoplasm. We observed that the leucine-rich domain of RGNEF is critical for its interaction with TDP-43 and localization in micronuclei. Finally, we described that micronuclei-like structures can be found in brain and spinal cord of ALS patients. This work is the first description of protein inclusion formation within micronuclei which also is linked with a neurodegenerative disease. The formation of TDP-43 inclusions within micronuclei induced by metabolic stress is a novel mechanism of protein aggregate formation which may have broad relevance for ALS and other neurodegenerative diseases.

## Introduction

Amyotrophic lateral sclerosis (ALS) is a progressive neurodegenerative disorder in which upper and lower motor neuron death leads to the loss of voluntary muscle function and death within 2–5 years of symptom onset^1^. The cause of the disease is unknown; however, there is increasing consensus that perturbations in RNA metabolism are key to the disease process^2,3^.

The formation of protein inclusions is a hallmark of the disease and includes RNA-binding proteins such as TAR DNA Binding Protein (TDP-43), Fused in Sarcoma/ Translocated in Liposarcoma (FUS/TLS), TATA-Box Binding Protein Associated Factor 15 (TAF 15), Ewing Sarcoma Breakpoint Region 1 (EWS), RNA Binding Motif Protein 45 (RBM45), Heterogeneous Nuclear Ribonucleoprotein A1 and A2/B1 (hnRNPA1 and hnRNPA2B1), and Rho Guanine Nucleotide Exchange Factor (RGNEF)^4–13^. Of these proteins, TDP-43 is the most extensively studied ALS-associated RNA-binding protein. There are multiple hypotheses for the mechanism by which TDP-43 forms pathological aggregates in ALS. Of these, one of the most accepted hypotheses is that the alteration of the dynamics and function of stress granules containing TDP-43 facilitates the nucleation and build-up of TDP-43 inclusions^14–19^. Additionally, the propensity of TDP-43 to form aggregates is exacerbated by mutations described in ALS patients^20–22^.

The study of the role of oxidative and osmotic stress in cells has been crucial to understand the mechanism of formation of TDP-43 aggregates and its relationship with stress granules^23,24^. Other types of cellular stress that might be relevant in the pathogenesis of ALS remain relatively unexplored. For example, dysmetabolism has been associated with neurodegenerative diseases^25^ and metabolic alterations have been repeatedly reported in ALS patients^26–30^. However, the contribution of cellular metabolic stress^31^ to ALS and its potential association with mechanisms of protein aggregation formation is currently unknown.

Previously, our group reported the existence of RGNEF-containing neuronal cytoplasmic inclusions (NCIs) in the motor neurons of ALS patients that co-aggregate with TDP-43^11,12^. RGNEF is a 190 kDa bifunctional guanine exchange factor/RNA binding protein that acts as a cellular survival factor under stress conditions^12,32^. RGNEF’s extensive localization in NCIs in ALS suggests it plays a critical role in the disease.

In this study, we demonstrate that under metabolic stress induced by lactate, both the leucine-rich (LeuR) domain of RGNEF and endogenous RGNEF co-localize with endogenous TDP-43 within micronuclei. Micronuclei are small extranuclear chromatin bodies surrounded by a nuclear envelope and positioned outside of the adjacent primary nucleus, that originate from chromosome segregation errors during mitosis^33,34^. Also, we observe that RGNEF’s LeuR domain is important for the co-localization and interaction between RGNEF and TDP-43. Notably, we show that endogenous TDP-43 forms inclusions inside micronuclei that co-aggregate with endogenous RGNEF but not with endogenous FUS/TLS or proteins not associated with ALS. Moreover, we show that micronuclei lose nuclear membrane integrity releasing TDP-43 aggregates to the cytoplasm. We also demonstrate the presence of TDP-43 positive micronuclei-like structures in tissue from ALS patients. Our results suggest that the formation TDP-43 aggregates within micronuclei is a potential novel mechanism of inclusion formation that could be relevant to ALS pathology.

## Results

### The LeuR domain of RGNEF localizes in TDP-43-positive micronuclei after metabolic stress

**Figure 1:**
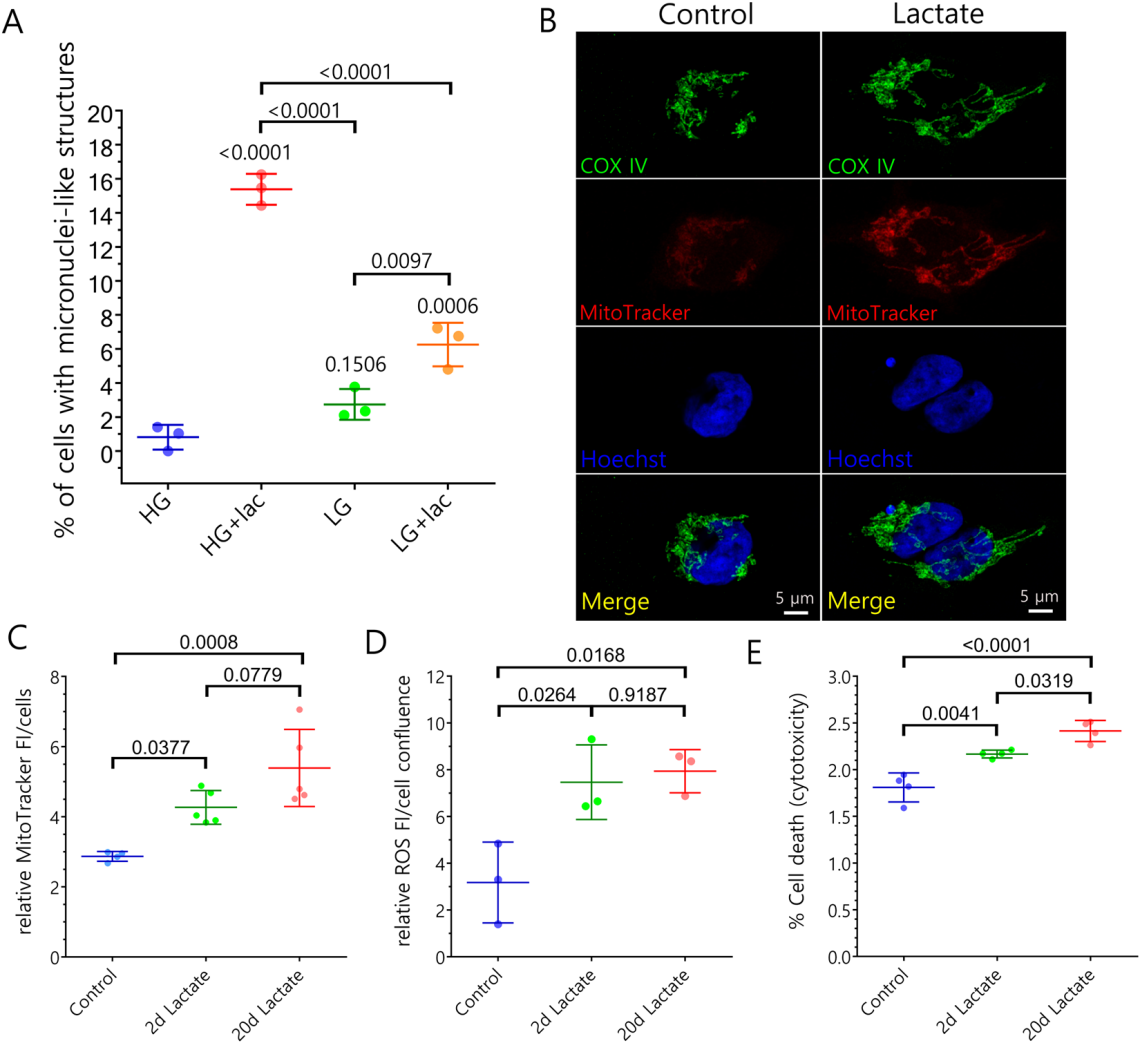
Metabolic stress induces the formation of micronuclei-like structures in HEK293T cells. (**A**) Quantification of micronuclei-like structures formation under different conditions (HG = high glucose; LG = low glucose; lac = lactate). Data are presented as mean ± SD and p values are indicated (two-way ANOVA analysis with Tukey post-hoc test; n = 3). (**B**) Representative confocal images of HEK293T cells under control or metabolic stress conditions during 20 days showing the difference in the fluorescence of MitoTracker Red CM-H_2_XRos as measure of the changes in mitochondrial membrane potential. Staining for COX IV was used as mitochondrial marker. Hoechst was used as nucleic acid marker. (**C**) Quantification of the fluorescence intensity (FI) of the oxidized form of MitoTracker Red CM-H_2_XRos under control conditions and after 2 and 20 days of metabolic stress induced by lactate. Only the oxidized form of MitoTracker is fluorescent and retained in the mitochondria as measure of the its metabolic activity. Data are presented as mean ± SD and p values are indicated (ANOVA analysis with Tukey post-hoc test; n control = 4; n treatments = 5). (**D**) Quantification of reactive oxygen species (ROS) analyzing the fluorescence intensity (FI) of oxidized intracellular carboxy-H_2_DCFDA after incubation under control conditions and after 2 and 20 days of metabolic stress induced by lactate. Only the oxidized form of carboxy-H_2_DCFDA is fluorescent and accounts for the amount of intracellular ROS under the studied conditions. Data are presented as mean ± SD and p values are indicated (ANOVA analysis with Tukey post-hoc test; n = 3). (**E**) Cytotoxicity assay showing the percentage of dead cells under control conditions and after 2 and 20 days of metabolic stress induced by lactate. Data are presented as mean ± SD and p values are indicated (ANOVA analysis with Tukey post-hoc test; n = 4).

In order to analyze the effect of metabolic stress on TDP-43 cellular localization and its potential for protein aggregate induction, we tested several conditions including high and low glucose in the presence or absence of lactate. Compared to the control condition (25 mM glucose media; high glucose), we observed an increase of 7.7-fold of globular cytoplasmic structures containing nucleic acids and the nuclear RNA-binding protein TDP-43, which resemble micronuclei in HEK293T cells incubated with 30 mM lactate in low glucose media (0.6 mM glucose) and 3.4-fold in cells only in low glucose media. Notably, we observed a more robust increase in the number of these micronuclei-like structures when cells were incubated with 30 mM lactate in high glucose media compared to control (18.8-fold increase) (Fig. 1A). Under this condition, in which the lactate concentration is in the same range of the glucose concentration of the media, we observed an increase of mitochondrial metabolism (Fig. 1B,C), increase in reactive oxygen species (ROS) concentration (Fig. 1D) and a mild cytotoxic effect over HEK293T cells (Fig. 1E), all consistent with a moderate stress condition. The stress under this condition is probably generated because gluconeogenesis is halted due to the high glucose concentration which may induce overload of the mitochondrial metabolic activity by lactate.

**Figure 2:**
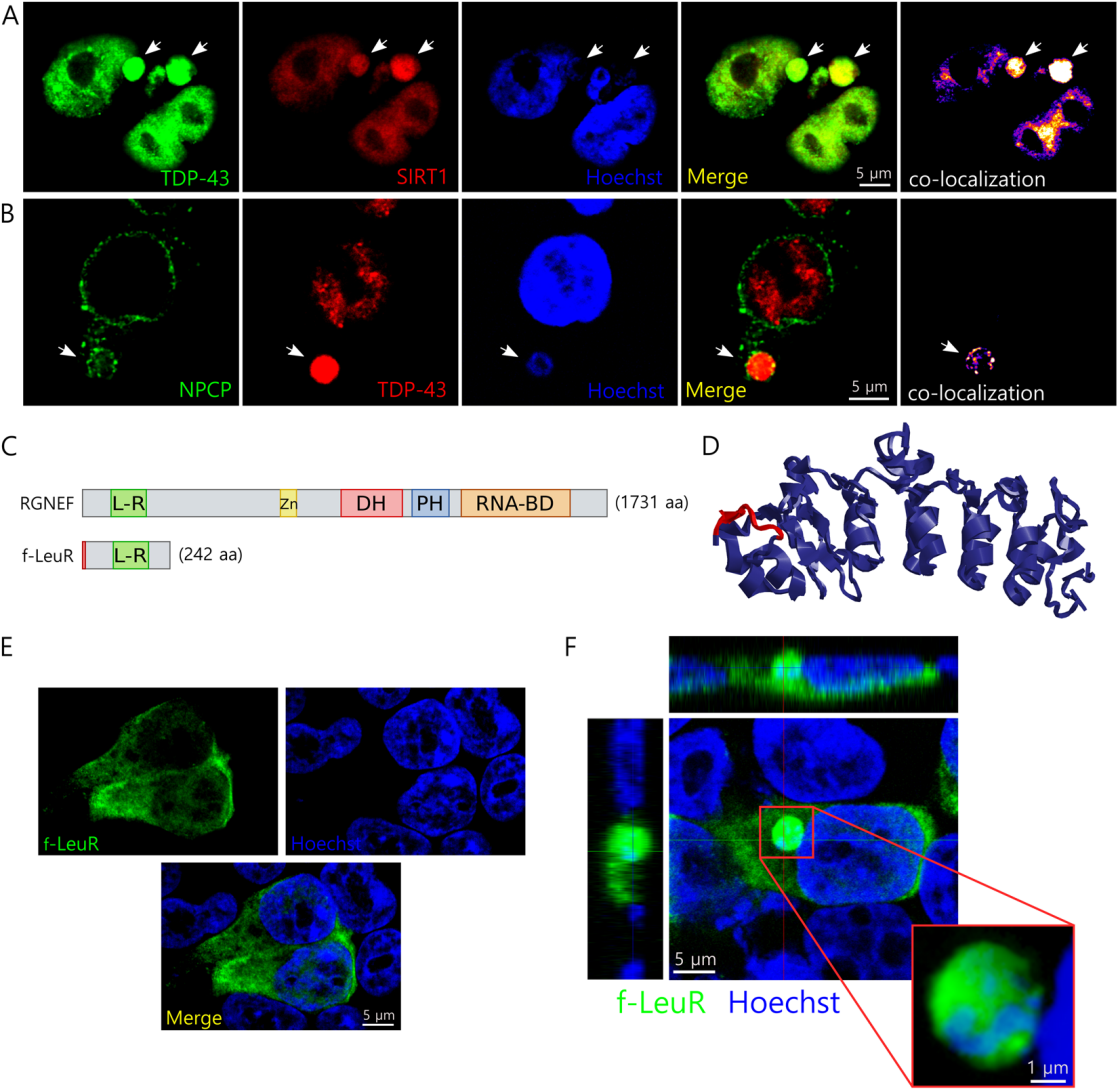
f-LeuR localizes within micronuclei after metabolic stress. (**A**,**B**) Representative confocal images showing the characterization of the micronuclei (white arrows) generated under metabolic stress in HEK293T cells using different nuclear markers. Immunofluorescence primary antibodies used were: anti-flag (**A**), anti-TDP-43 (**A**,**B**), anti-sirtuin 1 (SIRT1) (**A**) and anti-NPCP (**B**). Hoechst was used as nucleic acid staining. Scales are indicated in the images. (**C**) Schematic of the flag-tagged RGNEF construct containing the LeuR domain (f-LeuR) used in the experiments compared with full length RGNEF. Abbreviations: L-R = LeuR domain; Zn = cysteine-rich Zinc binding domain; DH = Dbl homology domain; PH = Pleckstrin homology domain; RNA-BD = RNA-binding domain. (**D**) *In silico* prediction of f-LeuR structure using I-Tasser software (flag is highlighted in red). (**E**) Representative confocal showing the cellular localization of f-LeuR in basal conditions in HEK293T cells. (**F**) Representative confocal image of HEK293T cells showing a micronucleus (enlarge on inset) containing f-LeuR and nucleic acids after metabolic stress. Orthogonal sections are shown. f-LeuR was detected using mouse anti-flag antibody. Hoechst was used as nucleic acid staining. Scales are indicated in the images.

To confirm if these structures were actually micronuclei, we analyzed whether they contained nuclear markers together with nucleic acids. We observed that the structures formed after metabolic stress co-localized with Sirtuin 1, a nuclear deacetylate enzyme; TDP-43, and nucleic acids (Fig. 2A). Also, we observed localization of TDP-43, nuclear pore complex proteins (NPCP), and nucleic acids (Fig. 2B) in the same structures, confirming they were micronuclei. These structures didn’t show presence of the cytoplasmic protein poly-A binding protein (Supplementary Figure S1A) or an enrichment of ALS-linked protein SOD1 that is present in both nucleus and cytoplasm (Supplementary Figure S1B). Additionally, we also observed the formation of micronuclei after metabolic stress in the neuronal cell line SH-5YSY (Supplementary Figure S1C).

**Figure 3:**
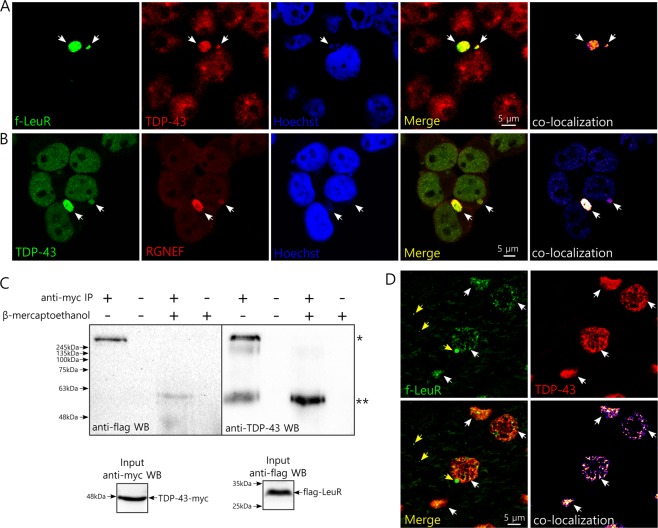
TDP-43 co-localizes with f-LeuR and endogenous RGNEF within micronuclei and interacts *in vitro* and co-localizes *in vivo* with f-LeuR. (**A**,**B**) Representative confocal images of HEK293T cells showing co-localization (white arrows) of endogenous TDP-43 with f-LeuR (A) or endogenous RGNEF (B) within micronuclei after cellular metabolic stress using lactate. (**C**) IP of TDP-43-myc after crosslinking using DTSSP on protein lysate from HEK293T cells expressing f-LeuR and TDP-43-myc. WB was performed for detecting flag and then TDP-43 after stripping. Input controls are showed. β-mercaptoethanol was used to dissociate the crosslinked complex (* and ** mark electrophoretic shifts of approx. 440 kDa and 60 kDa, respectively). (**D**) Schematic of scAAV-9-LeuR virus and representative confocal images showing the extensive co-localization (white arrows) between LeuR and TDP-43 in brain of rats 4 weeks after the injection with the virus that express flag-LeuR in neurons under SYN1 promoter (yellow arrows indicate granular L-rich that doesn’t co-localize with TDP-43). The proteins were detected using goat anti-flag and rabbit anti-TDP-43 antibodies.

Since we observed the presence of TDP-43 in micronuclei after metabolic stress we decided to evaluate whether these TDP-43 -containing micronuclei were also enriched with endogenous full length RGNEF or an RGNEF fragment containing its LeuR domain in HEK293T cells. Leucine-rich (LeuR) domains have been described to be highly relevant for protein-protein interactions^35^. Because of this we hypothesize that the LeuR region of RGNEF is critical for the aggregate formation of RGNEF in motor neurons of ALS patients. To study the subcellular localization of RGNEF’s LeuR under stress we made a construct expressing a flag-tagged version of the first 242 amino acids of RGNEF containing the LeuR domain (f-LeuR; Fig. 2C,D). The f-LeuR construct was expressed in HEK293T cells and incubated with 30 mM lactate in high glucose media to induce metabolic stress. Under metabolic stress, f-LeuR showed a change in its localization pattern, going from mainly cytoplasmic homogenous localization under basal conditions (Fig. 2E), similar to full length RGNEF^12,36^, to be observed highly concentrated in micronuclei structures (Fig. 2F). Interestingly, under metabolic stress, we observed micronuclei containing endogenous TDP-43 highly enriched with f-LeuR. In some cases, we were able to detected cells containing f-LeuR only in micronuclei (Fig. 3A). TDP-43 enriched micronuclei were also highly enriched in endogenous RGNEF despite the low levels of endogenous RGNEF in HEK293T cells^32^ (Fig. 3B). It is worth noting that lactate increases the translocation of RGNEF to the nucleus in HEK293T cells (Supplementary Figure S1D). This effect of lactate may explain why RGNEF is enriched in micronuclei. As control for the specificity of LeuR domain over the localization of RGNEF in micronuclei, we performed an experiment expressing an RGNEF construct lacking the LeuR region (RGNEF-∆LeuR-myc; Supplementary Figure S2A). We observed that this construct was unable to localize in micronuclei under metabolic stress induced by lactate (Supplementary Figure S2B).

Our results indicate that metabolic stress induces the formation of micronuclei in HEK293T and SH-SY5Y cells and show that in HEK293T cells the micronuclei are enriched with f-LeuR, endogenous TDP-43 and endogenous RGNEF. These experiments also suggest that the LeuR region of RGNEF is necessary for its localization in micronuclei following metabolic stress.

### TDP-43 interacts *in vitro* and co-localizes *in vivo* with f-LeuR

The co-localization of RGNEF’s LeuR domain with TDP-43 in micronuclei suggests that RGNEF and TDP-43 may interact through RGNEF’s LeuR domain and be part of a protein complex. This idea is consistent with our previous observation showing co-immunoprecipitation between TDP-43 and full-length RGNEF^11^. Here, we transfected HEK293T cells with plasmids expressing TDP-43-myc and f-LeuR and then we crosslinked the proteins using DTSSP. We then performed immunoprecipitation using anti-myc antibody. Interestingly, after Western Blotting and probing with anti-flag antibody, we were able to detect the presence of f-LeuR in a protein complex with an electrophoretic shift of approximately 440 KDa (Fig. 3C, left panel). When we treated the samples with the reducing agent β-mercaptoethanol, which dissociates the crosslinking between the proteins, that complex was eliminated and a new band appeared at approximately 60 KDa. After stripping, we confirmed the presence of TDP-43 in those complexes using antibody against this protein (Fig. 3C, right panel). As control, we performed this experiment expressing RGNEF-∆LeuR-myc. Under the same experimental conditions, we were unable to observe the immunoprecipitation of a high molecular weight complex containing TDP-43 (Supplementary Figure S2C).

To analyze if this *in vitro* interaction has an *in vivo* correlate, we injected rats with rAAV9 that expressed f-LeuR or GFP as control (Fig. 3D and Supplementary Figure S3D, E,F). We observed a high degree of co-localization between endogenous TDP-43 and f-LeuR in brain neuronal cells (Fig. 3D). This observation strongly suggests that RGNEF and TDP-43 are also able to interact through RGNEF’s LeuR domain under physiological conditions *in vivo*.

These results indicate that the LeuR domain of RGNEF may be critical for the formation of the RGNEF-TDP-43-containing aggregates observed in ALS patients.

### TDP-43 forms aggregates inside micronuclei

**Figure 4:**
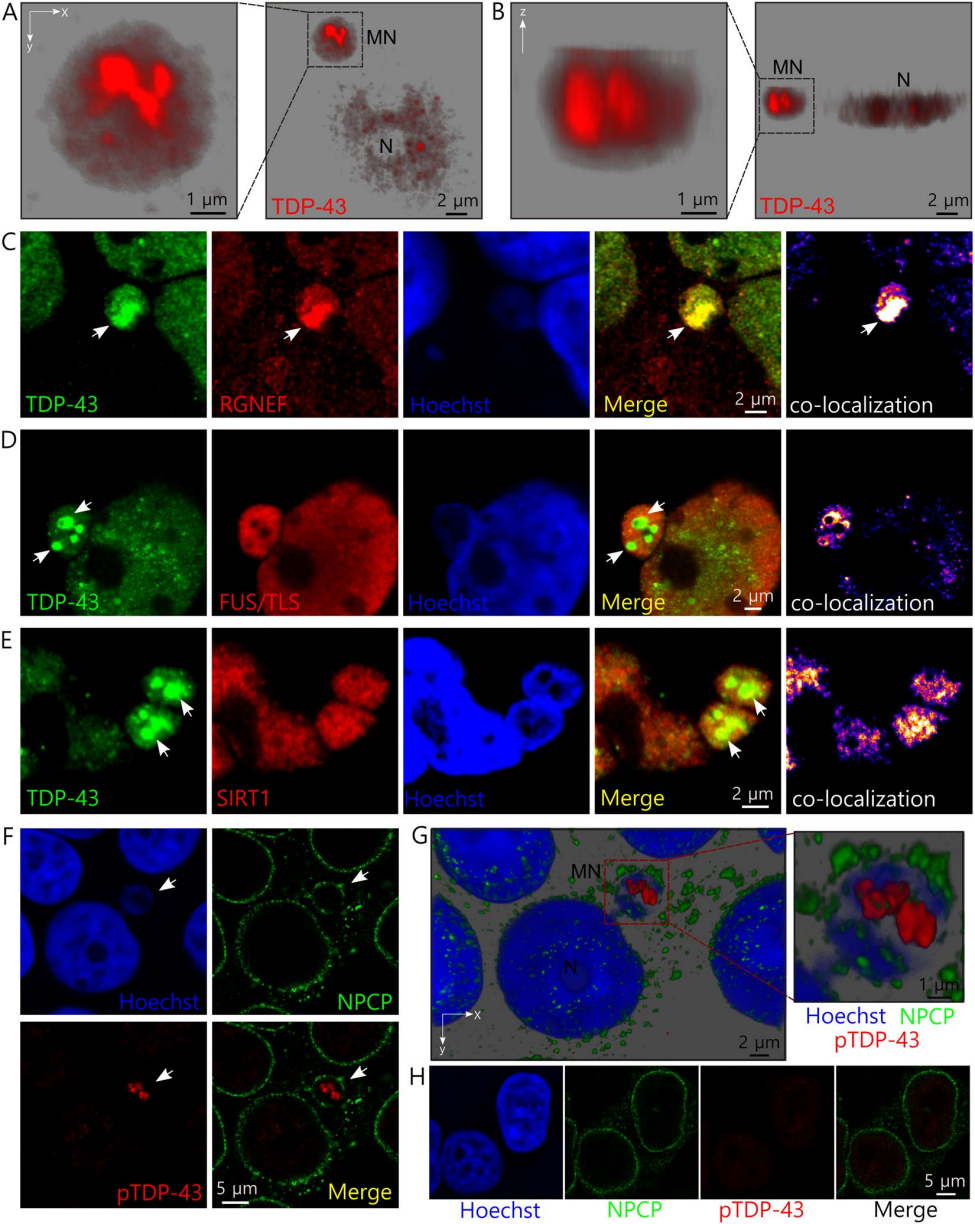
TDP-43 forms inclusions within micronuclei induced by metabolic stress which co-aggregate with endogenous RGNEF in HEK293T cells. (**A**,**B**) 3D reconstruction from confocal set of images of a HEK293T cell showing TDP-43 aggregates within a micronucleus after cellular metabolic stress using lactate. (**A**) Shows xy axis view and (**B**) shows z axis view of the cell. MN = micronucleus and N = nucleus. (**C**) Co-aggregation between endogenous TDP-43 and endogenous RGNEF within a micronucleus after metabolic stress in HEK293T cells (white arrows). (**D**,**E**) Absence of aggregate formation for FUS/TLS (**D**) and sirtuin 1(SIRT1) (**E**) within micronuclei in HEK293T cells. TDP-43 inclusions are indicated (white arrows). (**F**) TDP-43 aggregates within micronuclei in HEK293T after metabolic stress cells are immunoreactive for phospho(409/410)-TDP-43 antibody which only stain insoluble inclusions. (**G**) 3D reconstruction from confocal set of images of the cell in (**F**) showing the phospo(409/410)-TDP-43 positives aggregates. (**H**) Control HEK293T cells showing absence of immunoreactive for phospho(409/410)-TDP-43 antibody. Immunofluorescence primary antibodies used were anti-TDP-43, anti-RGNEF, anti-FUS/TLS, anti-sirtuin 1 (SIRT1) and anti-NPCP. Hoechst was used as nucleic acid staining. Scales are indicated in the images.

Besides the enrichment of TDP-43 and RGNEF in micronuclei formed after metabolic stress, we noted the formation of TDP-43 protein inclusions inside these structures. Using 3D rendering of confocal images, we observed that TDP-43 inclusions formed within micronuclei had typically 1–3 µm of diameter filling a substantial volume of the micronuclei (Fig. 4A,B; Supplementary Video S1). In general, inclusions were ovoidal (Fig. 4A,B) or globular (Figure S3A), and sometimes elongated resembling skein-like aggregates (Supplementary Figure S3B). Interestingly, we observed that TDP-43 inclusions within micronuclei co-aggregated with endogenous RGNEF (Fig. 4C), but not with FUS/TLS (Fig. 4D), or sirtuin 1, a protein that is not associated with ALS (Fig. 4E). We also found TDP-43 aggregates within micronuclei induced by metabolic stress in SH-SY5Y cells (Supplementary Figure S3C), which didn’t co-aggregate with FUS/TLS (Supplementary Figure S3D). Notably, we observed that the aggregates within micronuclei in HEK293T cells were immunoreactive for anti-phospho(409/410)-TDP-43, a specific antibody that stains insoluble TDP-43 inclusions^37,38^ (Fig. 4F,G). The immunoreaction using this antibody was negative for HEK293T cells under control conditions (Fig. 4H).

These results suggest that TDP-43 together with RGNEF forms inclusions within micronuclei and that micronuclei facilitate the co-aggregation between TDP-43 and RGNEF, but not with FUS/TLS. Also, our results suggest that the TDP-43 aggregates observed within micronuclei are pathological in nature.

### Micronuclei undergo a disruptive process and are present in ALS tissues

**Figure 5:**
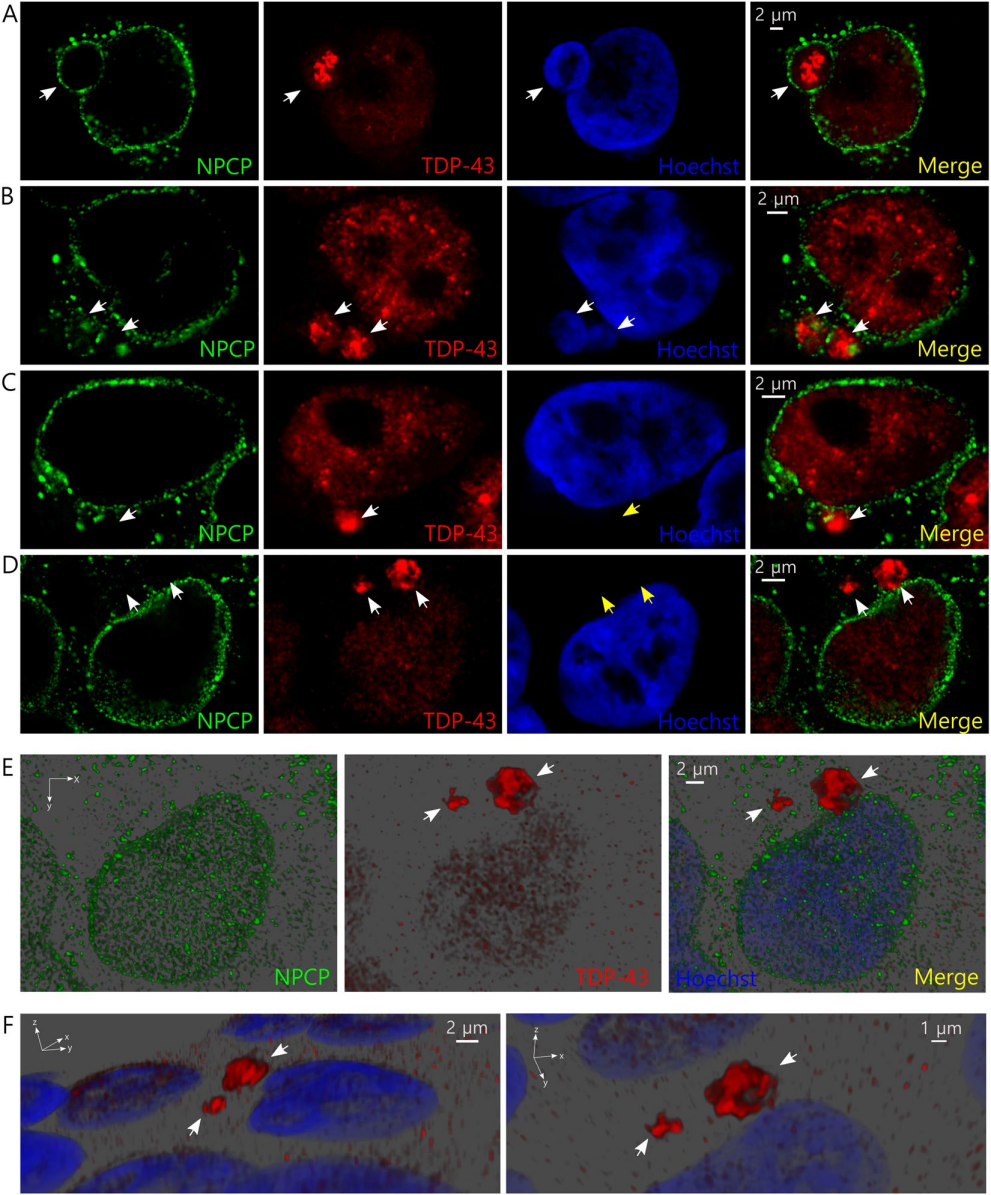
Micronuclei are disrupted probably releasing TDP43 aggregates to the cytoplasm in HEK293T cells. (**A**–**D**) Representative confocal images showing different stages of micronuclei disruption in cells after metabolic stress in HEK293T cells: intact micronucleus (continuous) (**A**), partial disruption (discontinuous) (**B**), absence of nuclear membrane proteins and detectable nucleic acids (collapsed) (**C**), and collapsed micronucleus with observation of massive cytoplasmic aggregates (**D**). Micronuclei containing TDP-43 aggregates are indicated by white arrows. Absence of nucleic acids in (**C**) and (**D**) is indicated by yellow arrows. (**E**,**F**) 3D reconstruction from confocal set of images of the cell presented in D showing the massive cytoplasmic aggregate formed after lactate-induced metabolic stress. (**E**) Shows the xy axis and (**F**) shows to different xyz angles that allows detailed observation of the aggregates (white arrows) shape and their position relative to the nucleus.

Micronucleus disruption has been previously described as the loss of micronuclei envelope permeability and its collapse implying the cessation of nuclear functions^39^. This may imply the release of the material previously in the micronuclei to the cytoplasm. To evaluate if a similar phenomenon occurs in HEK293T cells under metabolic stress, we analyzed the integrity of the nuclear membrane in cells containing TDP-43 inclusion-positive micronuclei after metabolic stress. Interestingly, we observed micronuclei with intact membranes (continuous membrane) (Fig. 5A) and also, micronuclei at different stages of the disruptive process, showing partial (discontinuous membrane) (Fig. 5B) or complete loss of the nuclear membrane marker (collapsed membrane) plus absence of detectable nucleic acids (Fig. 5C,D). Notably, between some of the cells with collapsed micronuclei we were able to also observe massive TDP-43 cytoplasmic aggregates (Fig. 5D,E,F). These observations support the idea that TDP-43 aggregates are likely released to the cytoplasm from disrupted micronuclei (Supplementary Figure S3E).

**Figure 6:**
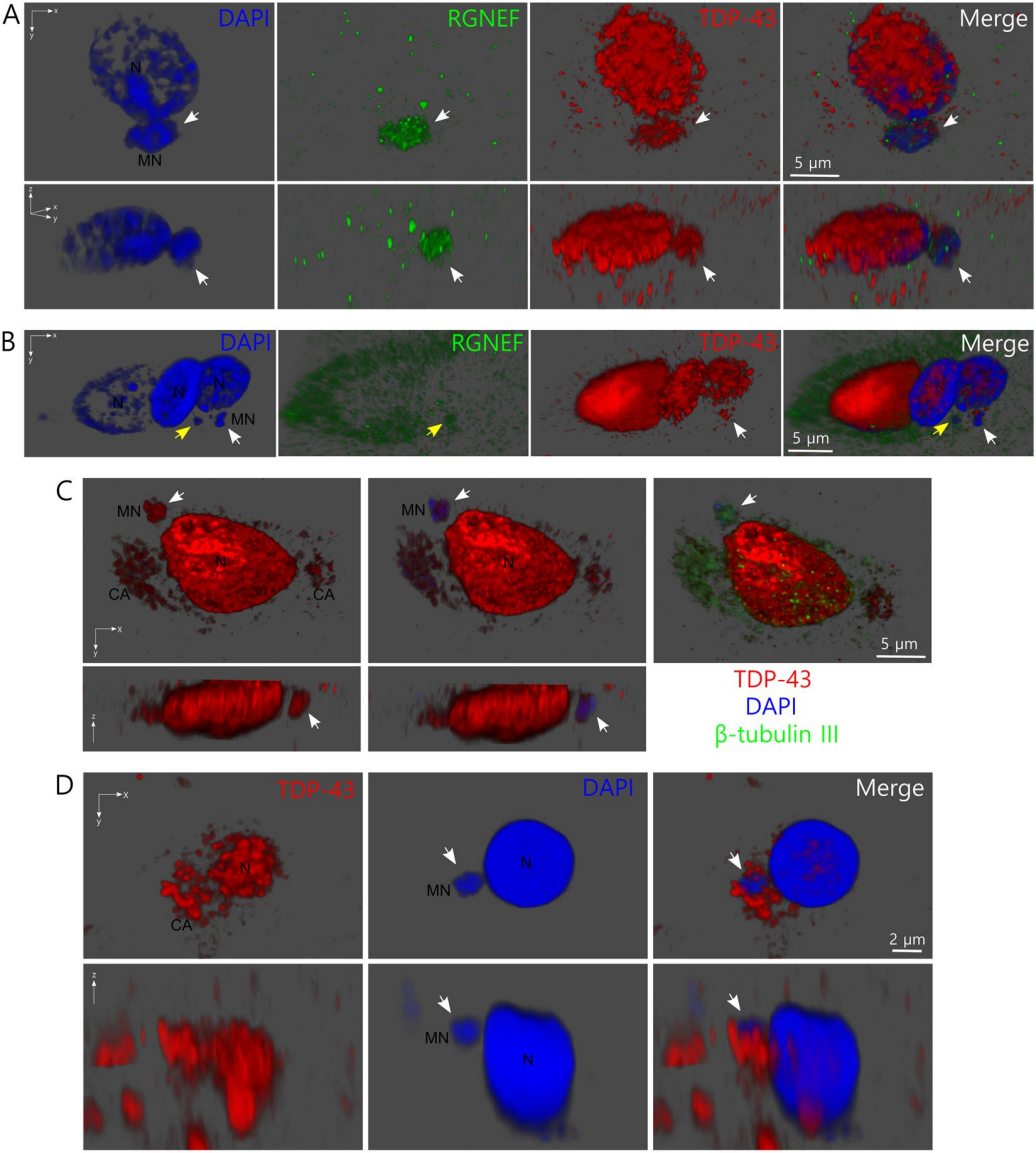
Micronucleus-like structures are observed in tissue from ALS patients. (**A**) 3D reconstruction from confocal set of images of a cell showing a micronucleus-like structure (white arrow) in hippocampus from ALS patient number 1. The upper panels show xy axis plane for DAPI staining, RGNEF, and TDP-43 detection. The lower panels show the lateral z axis planes of the images. Note that the micronucleus-like structure is positive for RGNEF, TDP-43 and nucleic acids. (**B**) 3D reconstruction from confocal set of images of a cell showing micronucleus-like structure (white arrow) in hippocampus from ALS patient number 2. The panels show xy axis plane for DAPI staining, RGNEF, and TDP-43 detection. The white arrow indicates a micronucleus-like structure positive for TDP-43 and nucleic acids. The yellow arrow indicates a micronucleus-like structure positive for RGNEF and nucleic acids. (**C)** 3D reconstruction from confocal set of images of a cell showing micronucleus-like structure (white arrow) in hippocampus from ALS patient number 3. The upper left panel shows xy axis plane for TDP-43 detection, the middle upper panel shows also DAPI staining and the upper right panel shows additionally the detection of β-tubulin III. The lower panels show the z axis planes for the TDP-43 detection and TDP-43 plus DAPI detection. (**D**) 3D reconstruction from confocal set of images of a cell showing micronucleus-like structure (white arrow) in cervical spinal cord from ALS patient number 3. TDP-43 and nucleic acids (using DAPI) were detected. Upper and lower panels show xy and z planes of the cell, respectively. Abbreviations: MN = micronucleus-like structure; N = cell nucleus; CA = TDP-43 cytoplasmic aggregates.

Our findings *in vitro* suggested that micronuclei formation in cells may be a relevant pathogenic mechanism in neurodegenerative diseases such as ALS. This would suggest the presence of observable micronuclei in tissues of ALS patients. We performed immunohistochemical analysis of ALS patient tissues to determine the presence of micronuclei. Notably, we were able to find micronuclei-like structures in the hippocampus (Fig. 6A–C) and the spinal cord (Fig. 6D) of ALS patients, which were not observed in control samples (Supplementary Figures S3F,G). We were able to observe a micronucleus-like structure positive for both RGNEF and TDP-43 (Fig. 6A) and also in a β-tubulin III positive cell (Fig. 6C), findings that strongly support our observations *in vitro*.

These results, in combination with our studies *in vitro*, suggest that formation of micronuclei containing TDP-43 inclusions might be a new potential mechanism of aggregate formation in ALS and other neurodegenerative diseases with TDP-43 proteinopathies.

## Discussion

The understanding of the mechanisms involved in the formation and spread of protein aggregates in ALS and other neurodegenerative diseases is a highly relevant topic currently in biomedicine. The present study has investigated a potential novel mechanism of neurodegeneration-related protein aggregation. Additionally, these results provided a deeper understanding of the mechanism of the strong co-aggregation of TDP-43 with RGNEF, a novel protein in ALS pathology^11,12^.

In order to induce the formation of protein inclusions we evaluated how cellular metabolic stress induced by lactate affects the subcellular localization of a protein carrying only the LeuR region of RGNEF (f-LeuR), a domain broadly implicated in protein-protein interactions. Interestingly, we observed lactate-induced formation of micronuclei in two human cell lines; HEK293T and the neuronal-like cell line SH-SY5Y. We selected these dividing cellular models since micronuclei are generated only as consequence of errors in the mitosis^33,34^. The HEK293T cell line was our main cellular model because in addition it is not derived originally from cancer^40^. We didn’t observe micronuclei formation induction using osmotic and oxidative acute stress^32^.

Recent attention has been brought to micronuclei because of the study of massive chromosomal rearrangements known as chromothripsis^41^, especially in the cancer field^34^. However, despite neurodegeneration and cancer sharing several molecular aspects^42^, the study of micronuclei formation in neurodegeneration has been limited to reports outside the nervous system in Alzheimer’s disease (AD) and Parkinson’s disease (PD). Micronuclei have been described as potential biomarkers for neurodegeneration as they are observed in higher frequency in peripheral lymphocytes, skin fibroblasts, and buccal mucosa cells from AD patients and peripheral lymphocytes from PD patients^43^.

The micronuclei we observed were enriched with endogenous TDP-43 which co-localized with f-LeuR and endogenous RGNEF. These results suggested RGNEF’s LeuR is critical for the localization of full RGNEF in micronuclei under cellular metabolic stress. Our observation that a protein including the LeuR of RGNEF is part of a protein complex containing TDP-43, and also co-localizes *in vivo* with TDP-43, not only confirmed our previous observation about the interaction between TDP-43 and full length RGNEF^11^, but also indicated that the LeuR domain may be critical for the formation of the RGNEF-TDP-43-containing aggregates observed in motor neurons of ALS patients^11,12^.

A key finding of this study was that TDP-43 forms inclusions inside the micronuclei induced by lactate stress in culture cells. As has been described previously, TDP-43 can form aggregates due to the dysregulation of protein-protein^44–47^ or protein-RNA^48,49^ interactions. This suggests that the interior of the micronuclei may generate a microenvironment of altered protein/RNA homeostasis leading to an aberrant TDP-43 phase separation and consequent induction of aggregation. This process could be analogous to the current postulated mechanism in which stress granules become protein inclusions^14–19^. Remarkably, the disrupted micronuclei we observed in cultured cells show that TDP-43 aggregates inside micronuclei are likely released to the cytoplasm (Fig. 5D–F).

Evidence that supports our *in vitro* observations as a model of a pathophysiological phenomenon, comes from the observation of micronuclei-like structures in samples from three ALS patients, which were absent in neurologically healthy patients. We focused our study in hippocampus and spinal cord because both present TDP-43 pathology in ALS^50^ and have been described to have neurogenesis^51,52^. Our main interest was to search for micronuclei in neurons; however, we cannot discard the possibility that micronuclei-like structures are also present in other types of neural cells.

We hypothesize that micronuclei formation is likely an early event in the ALS pathogenic process, as in our model micronuclei are generated as a result of a stress condition and only after that we observed aggregate formation. This could explain why we observed a relatively low incidence of micronuclei-like structures in the brain or spinal cord from ALS patients with advanced stages of the disease. A comprehensive quantitative analysis of micronuclei in early and late stage cohorts of ALS will illuminate this possibility, but this is beyond the scope of the current study.

In summary, this work shows that the LeuR domain of RGNEF may be critical for its co-aggregation with TDP-43 but most notably that the formation of TDP-43 inclusions within micronuclei could be one of the mechanisms of aggregate formation in neurodegenerative diseases such as ALS. Our observations open the door for a new fascinating field of study where the analysis of micronuclei formation and dynamics could be implicated in the pathogenesis of neurodegenerative diseases.

## Methods

### Cell lines

HEK293T and SH-5YSY cells (ATCC) were maintained in 25 mM glucose, 1 mM pyruvate Dulbecco’s modified Eagle’s medium (Gibco - Life technologies) containing 100U/ml penicillin, 100U/ml streptomycin (Gibco - Life technologies), 5 µg/ml plasmocin (InvivoGen), and 10% fetal bovine serum (Gibco - Life technologies).

### Human samples

Post-mortem frozen and formalin-fixed, paraffin-embedded tissues were collected as part of the ALS protocol at London Health Sciences Centre (London, Ontario, Canada). Ethics review and approval was granted by The University of Western Ontario Research Ethics Board for Health Sciences Research Involving Human Subjects (HSREB - Protocol #103735) for the use of brain and spinal cord tissues and for access to medical records for research purposes. Informed consent was obtained from all participants. All research was performed in accordance with relevant guidelines/regulations. Three sporadic ALS cases without known mutations in the coding sequence of SOD1, FUS/TLS and TDP-43 and without C9ORF72 expanded repeats, and three neurologically healthy controls were analyzed in this study.

### Rats

Female SAS Sprague-Dawley (Charles River), 12 weeks old and 250 g of weight, were used for the brain intraventricular injection experiment. All procedures involving animals, surgeries, and animal maintenance were in accordance with the Canadian Council for Animal Care and the University Council on Animal Care guidelines for research. Ethics review and approval was granted by the Animal Care Committee of The University of Western Ontario (Protocol #2017-035).

### Antibodies

See Supplementary Table S1.

### Cloning

For the generation of TDP-43 and LeuR constructs PCR reactions were performed using Phusion High-Fidelity DNA Polymerase (Thermo Scientific) and the products were cloned into the vectors pcDNA 3.1/myc-His A (Invitrogen) or pcDNA 3.1 (Invitrogen) respectively. A flag tag was added in the 5′ extreme of the coding sequence of LeuR and full length RGNEF for later detection using anti-flag antibodies. The sequences for the self-complementary adeno-associated viruses (scAAVs) were designed as described in the Figure S3. The sequences from the 5′ ITR to the 3′ ITR-∆trs were fully synthetized and cloned into the pUC18 vector (GeneScript) originating the vectors pscAAV-GFP and pscAAV-L-rich. All constructs were confirmed by sequencing.

### Metabolic stress conditions

To induce metabolic, stress cells were incubated with 30 mM lactate (DL-Lactic acid sodium salt, Sigma-Aldrich) in 10% FBS DMEM containing 25 mM glucose (high glucose-HG) and 1 mM pyruvate or 0.6 mM glucose (low glucose-LG) media (glucose given by the FBS present in the media).

### Transfections of cells under metabolic stress

HEK293T cells maintained 7 days under metabolic stress (HG + lactate) or under control conditions (HG) were seeded onto coverslips previously treated with attachment factor (Gibco) in 6-well plates at 250,000 cells/ml 24 hours before transfection. Transfections were performed using Lipofectamine 2000 (ThermoFisher Scientific) at 70% of confluency using 2.5 µg of DNA in a ratio (µg) DNA:(µl) lipofectamine 1:2.5. Cells were maintained under metabolic stress for 48 hours after the transfection. Then, cells in the coverslips were fixed in 4% paraformaldehyde in PBS for 15 min and processed for immunofluorescence. For the cross-linking-immunoprecipitation experiment transfections were performed using the same protocol, but using 6-well plates without coverslips and without metabolic stress.

### Immunofluorescence (cells)

Coverslips of cells fixed with 4% paraformaldehyde were permeabilized with 0.2% Triton X-100 for 10 min. Aldehyde groups were quenched using 50 mM ammonium chloride for 30 min. Then, cells were blocked with 8% bovine serum albumin for 1 hour at room temperature. The following primary antibodies were used: mouse anti-myc, mouse anti-flag, rabbit anti-TDP-43, mouse anti-TDP-43, rabbit anti-RGNEF, rabbit anti-FUS/TLS, rabbit anti-Sirt1, mouse anti-Nuclear Pore Complex Protein (NPCP), rabbit anti-SOD1, mouse anti-Poly A Binding Protein (PABP), or mouse anti-COX IV. Primary antibodies were detected using secondary antibodies conjugated to Alexa Fluor 488 or Alexa Fluor 555. Nuclei were stained with Hoechst (1 µg/mL).

### Micronuclei quantification

After maintaining HEK293T cells in high and low glucose media in presence of absence of 30 mM lactate during 10 days, immunofluorescence detecting TDP-43 and staining with Hoechst was performed. TDP-43 and Hoechst positive micronuclei were quantified by triplicate under all the conditions described with an average of 91.08 + 3.24 cells per field. Data is presented as the percentage of cells containing at least one micronucleus over the total amount of cells.

### Mitochondrial metabolic activity analysis

To evaluate the mitochondrial metabolic activity under metabolic stress we used MitoTracker Red CM-H2XRos. This nonfluorescent chemical passively diffuses across the plasma membrane and becomes fluorescent and accumulates in active mitochondria after oxidation. After 2 or 20 days under metabolic stress, cells were incubated with 400 nM MitoTracker Red CM-H2XRos (Invitrogen) at 37 °C for 45 min. Then, the cells were fixed and processed for immunofluorescence. The data were presented as relative MitoTracker fluorescence intensity divided by the number of cells for normalizing.

### Detection of reactive oxygen species (ROS)

The generation of ROS under metabolic stress was studied using carboxy-H2DCFDA. This chemical is nonfluorescent until the acetate groups are removed by intracellular esterases, and oxidation by ROS occurs within the cell. The oxidized compound is largely retained intracellularly. After 2 or 20 days under metabolic stress, the cells were incubated with 20 µM carboxy-H2DCFDA (Invitrogen) at 37 °C for 1 hour. Then, cells were left in recovery for 2 hours and observed under the confocal microscope (green channel). To quantify specific fluorescence due to ROS activity, an autofluorescence control of HEK293T cells not treated with carboxy-H2DCFDA was used. Data was presented as relative ROS fluorescence intensity divided by the confluence of cells for normalization. The confluence was obtained by measuring the area cover by cells (observed by autofluorescence in the blue channel) using the ImageJ software.

### Cytotoxicity analysis

Cells were seeded in white 96-well plates at 8,000 cells/ml per well. The cytotoxicity was measured using the CytoTox-Glo™ Cytotoxicity Assay kit (Promega) according to the manufacturer’s protocol after 2 or 20 days under metabolic stress. This kit quantifies “dead-cell protease activity”, which is released from cells that have lost membrane integrity. To obtain the percentage of cell death, the values obtained after the stress condition or control were normalized against total protease activity obtained after cell lysis using digitonin.

### Cross-linking experiment

Cross-linking was performed incubating 500 µg of protein lysate of HEK293T cells transfected with pcDNA-flag-L-rich and pcDNA-TDP-43-myc with 3,3′-Dithiobis(sulfosuccinimidylpropionate) (DTSSP; Thermo Scientific) to a final concentration of 1.3 mM in PBS buffer pH 7.2. After 30 min at room temperature, the cross-linking was quenched by the addition of 1 M Tris, pH 7.5 to a final concentration of 37 mM.

### Immunoprecipitation and immunoblot

Immunoprecipitations were performed using the Dyanbeads Protein G Immunoprecipation Kit (Invitrogen) according the manufacturer’s instructions. After the cross-linking reaction, each sample was immunoprecipitated using 2 µg of mouse anti-myc (Cedarlane) or mouse IgG (Sigma-Aldrich) as control. A group of samples was treated with 50 mM β-mercaptoethanol (Sigma) at 100 °C for 5 min to dissociate the cross-linking between the proteins. Then, all samples were subjected to SDS–polyacrylamide gel electrophoresis, followed by immunoblotting using goat anti-flag. After the immunoblot with anti-flag, membranes were stripped using 0.1 M Gly/HCl pH 2.5 45 min at room temperature and then 1X PBS 1 M NaCl by 8 min at room temperature. After, immunoblot using rabbit anti-TDP-43 was performed.

### scAAVs generation and injection

Using the pscAAV-GFP and pscAAV-LeuR plasmids, the self-complementary adeno-associated viruses serotype 9 for neuronal-specific expression of GFP (scAAV9-GFP) and the LeuR region of RGNEF (scAAV9-LeuR) (Figure S3A) were produced to a yield of 1.5 × 1013 GC/ml and 1.6 × 1013 GC/ml respectively (Vector Biolabs). Eight rats (4 for each virus) were injected with the AAVs intraventricularly in the brain (injection site: Bregma: −0.8 mm; 1.4 mm bilateral from midline; 3.7 mm vertical). 3–4 weeks post-surgery rats were fixed by perfusion with 4% paraformaldehyde and the brain extracted for further processing. All the work with animals was performed following the National Institutes of Health guide for the care and use of Laboratory animals (NIH Publications No. 8023, revised 1978) and as approved by the Animal Use Subcommittee of the Western University Council on Animal Care.

### Immunofluorescence (tissues)

Archival paraffin-embedded sections of brain from ALS cases and brain from rats injected with the AVVs post-fixed and embedded in paraffin, were serially sectioned at 6 μm thickness. Tissue sections were deparaffinized using standard protocols and following antigen retrieval (10 mM sodium citrate, 0.05% Tween 20, pH 6), the sections were incubated with the primary antibodies: goat anti-GFP, goat anti-flag, rabbit anti-TDP-43, or mouse anti-β tubulin III. Primary antibodies were detected using secondary antibodies conjugated to Alexa Fluor 488 or Alexa Fluor 555. Nuclei were stained with DAPI (5 µg/mL) and tissue then were visualized by scanning confocal microscopy.

### Confocal imaging and 3D analysis

Cells and tissues from immunofluorescence experiments were visualized by scanning confocal microscopy (Leica TCS SP8). The 3D reconstruction analysis of set of confocal images were performed using the Leica 3D analysis tool from LAS X software.

### Co-localization images

Intensity Correlation Analysis^53^ using ImageJ software was performed to obtain the co-localization images. The co-localized pixels are shown as PDM (Product of the Differences from the Mean) images. PDM = (red intensity-mean red intensity) × (green intensity-mean green intensity). In the co-localization images, blue color indicates a low level of co-localization while yellow and white indicate a high degree of co-localization.

### Protein modeling

Molecular modeling was performed using I-Tasser software (http://zhanglab.ccmb.med.umich.edu/I-TASSER/), which predicts protein structure using modeling by iterative threading assembly^54,55^. The models were visualized using RasTop 2.2 (http://www.geneinfinity.org/rastop).

### Statistical analysis

The statistical analyses were performed with GraphPad Prism 8.3 software using one-way or two-way ANOVA with Tukey post-hoc analysis to obtain exact p values. All data used for graphics are presented in the Supplementary Tables S2–S5. All the analysis passed normality (Shapiro-Wilk test) and equal variance test (Brown-Forsythe test). The results of the normality test are presented in the Supplementary Table S6. Data were expressed as mean ± SD. Data was judged to be statistically significant when p < 0.05. For experiments carried out using culture cells, n of 1 represents and independent plate of cells under control or treatment condition.

## Supplementary information

Supplementary information

Supplementary video
