# Supplementary material for: TDP-43 aggregation inside micronuclei reveals a potential mechanism for protein inclusion formation in ALS
Source: Sci Rep. 2019 Dec 27;9:19928. doi: 10.1038/s41598-019-56483-y (PMC6934605; doi:10.1038/s41598-019-56483-y)
Supplement: Supplementary file 1 — Supplementary information [file 41598_2019_56483_MOESM1_ESM.docx]

**TDP-43 aggregation inside micronuclei reveals a potential mechanism for protein inclusion formation in ALS: Supplementary files**

Cristian A. Droppelmann^1*^, Danae Campos-Melo^1^, Alexander J. Moszczynski^1^, Hind Amzil^1^, and Michael J. Strong^1,2*^

^1^Molecular Medicine Group, Robarts Research Institute, Schulich School of Medicine & Dentistry, Western University, London, Ontario, Canada.

^2^Department of Clinical Neurological Sciences, Schulich School of Medicine & Dentistry, Western University, London, Ontario, Canada.

^*^Corresponding Authors: Dr. Cristian A. Droppelmann (cdroppel@uwo.ca) and Dr. Michael J. Strong (Michael.Strong@schulich.uwo.ca) (main contact)


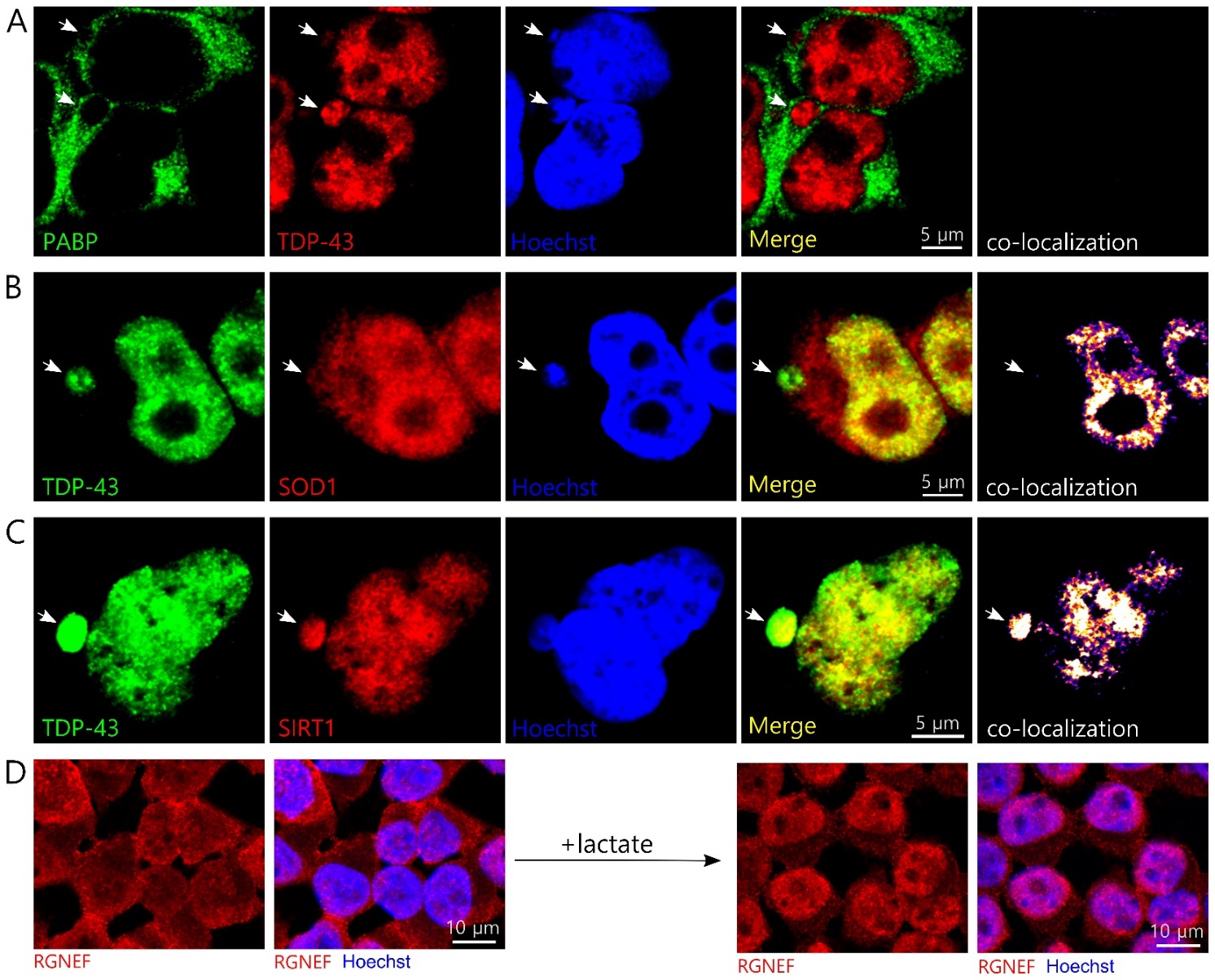


**Supplementary Figure S1.**

**A** and **B)** Representative confocal images of HEK293T cells showing the homogeneous localization of endogenous poly-A binding protein (PABP) (C) or endogenous super oxide dismutase 1 (SOD1) in the cytoplasm (D), while endogenous TDP-43 is observed within micronuclei after cellular metabolic stress using lactate (white arrows indicate micronuclei positives for TDP-43). There is no co-localization between PABP or SOD1 and TDP-43 in the micronuclei. **C)** Representative confocal images of SH-SY5Y cells showing co-localization of endogenous TDP-43 and SIRT-1 within micronuclei after cellular metabolic stress using lactate (white arrows). **D)** Representative confocal images of the cellular localization of endogenous RGNEF in HEK293T cells without and with lactate treatment. Metabolic stress induces a remarkable translocation of RGNEF from the cytoplasm to the nucleus.


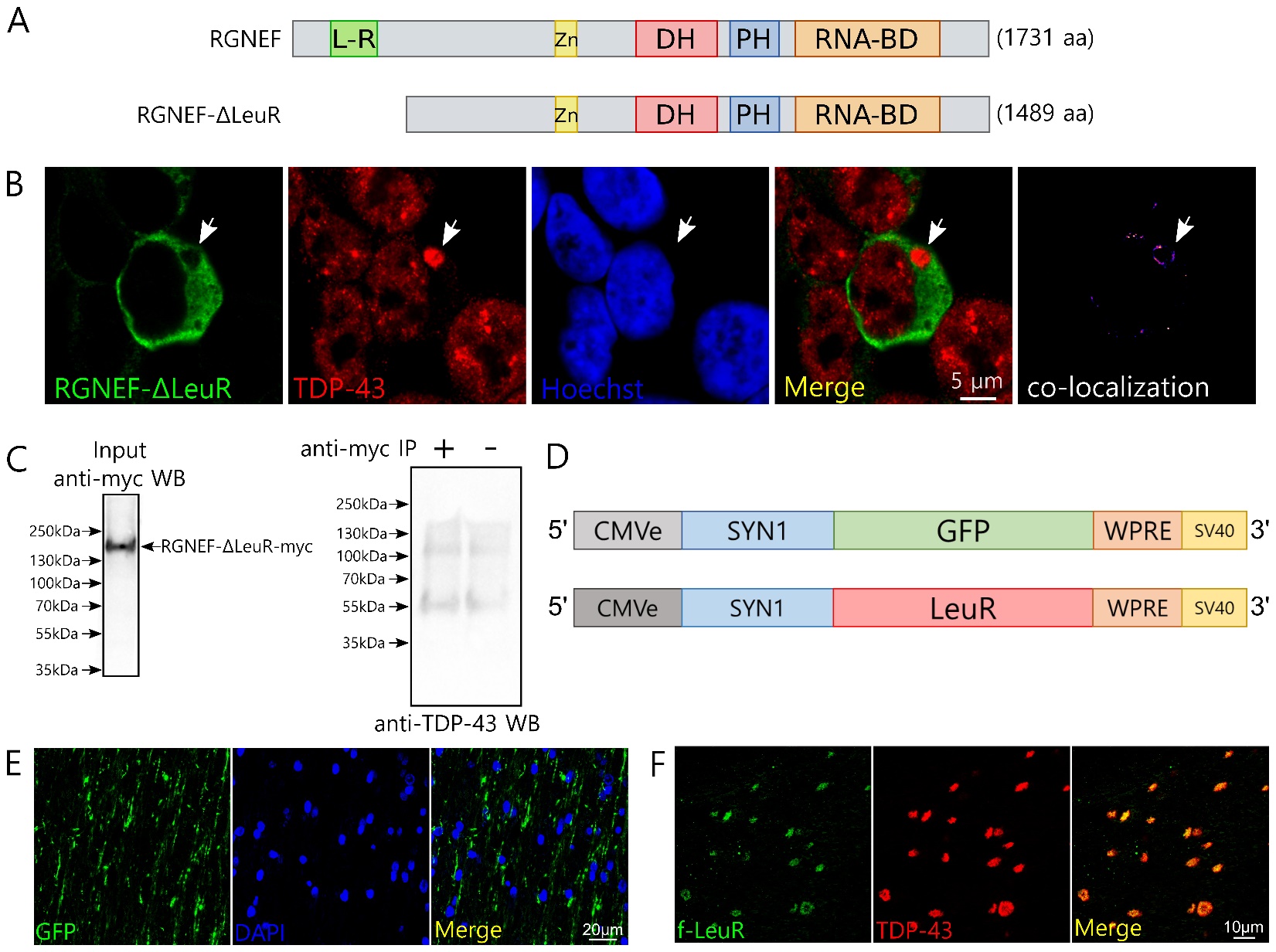


**Supplementary Figure S2.**

**A**) Schematic of the myc-tagged RGNEF construct without the LeuR domain used in the experiments compared with full length RGNEF. Abbreviations: L-R=LeuR domain; Zn=cysteine-rich Zinc binding domain; DH=Dbl homology domain; PH=Pleckstrin homology domain; RNA-BD=RNA-binding domain. **B**) Representative confocal images of HEK293T cells showing the homogeneous localization of RGNEF-∆LeuR-myc while endogenous TDP-43 is observed within micronuclei (white arrow) after cellular metabolic stress using lactate. There is not co-localization between RGNEF-∆LeuR-myc and TDP-43 in the micronuclei. Hoechst was used as nucleic acid staining. **C**) IP of RGNEF-∆LeuR-myc after crosslinking using DTSSP on protein lysate from HEK293T cells. WB was performed for detecting TDP-43 under not reducing conditions. It was not observed co-immunoprecipitation between RGNEF-∆LeuR-myc and TDP-43. Input control is shown.

**D)** Schematic of the coding and regulatory elements of scAAV-9 viruses used for the intraventricular injections on rats. The regulatory elements of scAAV-9 viruses used for the intraventricular injections on rats are: CMVe=cytomegalovirus enhancer sequence; SYN1= synapsin 1 promoter; GFP= green fluorescent protein coding region; LeuR= f-LeuR coding region; WPRE= Woodchuck hepatitis virus posttranscriptional regulatory element; SV40= SV40 late polyadenylation (LPA) signal plus SV40 LPA upstream element. **E)** Localization of GFP in the brain of rats 4 weeks after the injection. The protein was detected using goat anti-GFP antibody. Nuclei were stained with DAPI. **F)** Localization of f-LeuR in the brain of rats 4 weeks after the injection. The protein was detected using goat anti-flag antibody. Nuclei were marked using rabbit anti-TDP-43 antibody.


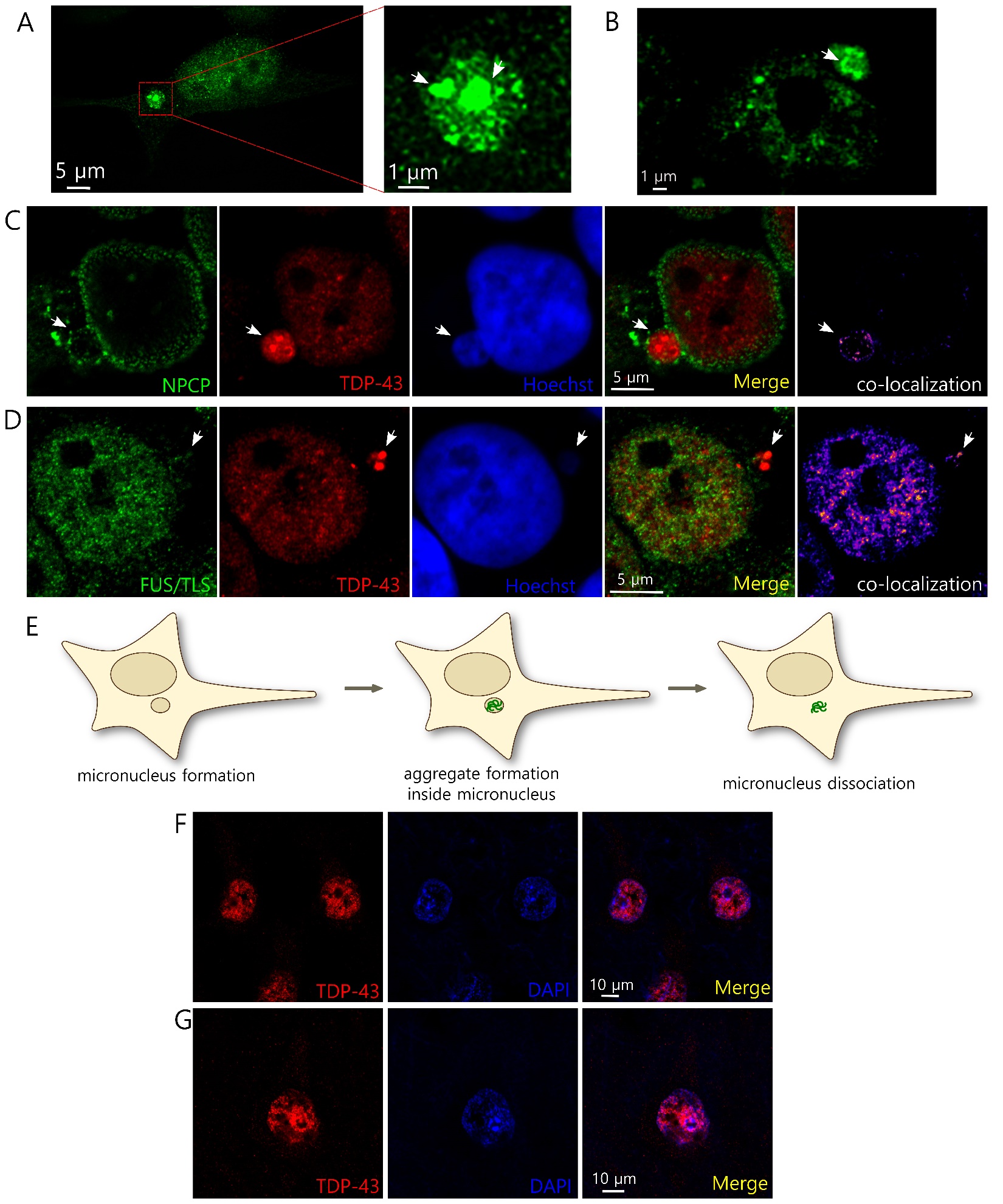


**Supplementary Figure S3.**

**A)** Confocal image showing globular TDP-43 aggregates inside a micronucleus after metabolic stress in HEK293T cells. **B)** Confocal image showing elongated TDP-43 aggregates inside a micronucleus after metabolic stress in HEK293T cells. **C** and **D**) Representative confocal images showing TDP-43 aggregates within micronucleus (white arrows) generated under metabolic stress in SH-SY5Y cells. Immunofluorescence primary antibodies used were: anti-NPCP (A), anti-FUS/TLS (B), and anti-TDP-43 (A and B). Hoechst was used as nucleic acid staining. Note that TDP-43 aggregates in the micronucleus do not co-aggregate with FUS/TLS. **E)** Schematic showing the model of how TDP-43 aggregates are released to the cytoplasm after the disruption of the micronucleus. **F** and **G**) Representative confocal images showing the localization of TDP-43 in the hippocampus region of the brain in two neurologically healthy patients. DAPI was used as nucleic acid staining. Scales are indicated in the images.

**Supplementary Video S1:** Animation of the 3D reconstruction showed in Figures 4A and B.

| **Supplementary Table S1**. List of antibodies used in this paper (IF = immunofluorescence; WB = Western blot) | | | |
| --- | --- | --- | --- |
| **Antibody** | **Company** | **Dilution used** | **Catalog number** |
| Anti-myc (mouse) | Cedarlane | 1:250 (IF) | CLX229AP |
| Anti-flag (mouse) | Sigma | 1:400 (IF) | F1804 |
| Anti-flag (goat) | Abcam | 1:400 (IF)  1:2,500 (WB) | ab1257 |
| Anti-TDP-43 (rabbit) | Proteintech | 1:500 (IF)  1:2,500 (WB) | 10782-2-AP |
| Anti-TDP-43 (mouse) | Proteintech | 1:500 (IF) | 60019-2-IG |
| Anti-phospho(409/410)-TDP-43 (rabbit) | Proteintech | 1:250 (IF) | 22309-1-AP |
| Anti-RGNEF (rabbit) | Abcam | 1:100 (IF) | ab122399 |
| Anti-RGNEF (goat) | MediMabs | 1:100 (IF) | MM-0193-P |
| Anti-FUS/TLS (rabbit) | Proteintech | 1:100 (IF) | 11570-1-AP |
| Anti-Sirt1 (rabbit) | Santa Cruz Biotechnology | 1:50 (IF) | sc-15404 |
| Anti-Nuclear Pore Complex Protein (NPCP) (mouse) | BioLegend | 1:500 (IF) | MMS-120P |
| Anti-SOD1(rabbit) | Enzo Life Sciences | 1:200 (IF) | ADI-SOD-100 |
| Anti-Poly A Binding Protein (PABP) (mouse) | Abcam | 1:500 (IF) | ab6125 |
| Anti-GFP (goat) | Abcam | 1:400 (IF) | ab6673 |
| Anti-COX IV (mouse) | ThermoFisher Scientific | 1:125 (IF) | K.473.4 |
| Anti-β tubulin III (mouse) | Sigma | 1:1,000 (IF) | T8660 |
| Anti-mouse Alexa 488 (donkey) | Invitrogen | 1:800 (IF) | A21202 |
| Anti-rabbit Alexa 555 (donkey) | Invitrogen | 1:800 (IF) | A31572 |
| Anti-goat Alexa 488 (donkey) | Invitrogen | 1:800 (IF) | A11055 |

| Supplementary Table S2. Micronuclei quantification data (HG=high glucose; LG= low glucose). | | | |
| --- | --- | --- | --- |
| Sample | **Number of cells** | **Number of cells with micronuclei** | **% of cells with micronuclei** |
| HG (1) | 96 | 1 | 1.041 |
| HG (2) | 71 | 1 | 1.408 |
| HG (3) | 91 | 0 | 0 |
| HG + lactate (1) | 97 | 15 | 15.463 |
| HG + lactate (2) | 97 | 14 | 14.432 |
| HG + lactate (3) | 80 | 13 | 16.250 |
| LG (1) | 95 | 2 | 2.105 |
| LG (2) | 85 | 2 | 2.352 |
| LG (3) | 106 | 4 | 3.773 |
| LG + lactate (1) | 104 | 5 | 4.807 |
| LG + lactate (2) | 97 | 7 | 7.216 |
| LG + lactate (3) | 74 | 5 | 6.756 |

| Supplementary Table S3. Mitochondrial activity quantification data. | | | |
| --- | --- | --- | --- |
| Sample | **sum pixels** | **cell number** | **pixels/cell** |
| Control (1) | 35064557 | 131 | 267668.374 |
| Control (2) | 44301740 | 150 | 295344.933 |
| Control (3) | 42146975 | 141 | 298914.716 |
| Control (4) | 46543868 | 163 | 285545.202 |
| Lactate 2d (1) | 41062782 | 107 | 383764.317 |
| Lactate 2d (2) | 68584602 | 176 | 389685.239 |
| Lactate 2d (3) | 75690481 | 155 | 488325.683 |
| Lactate 2d (4) | 58287773 | 131 | 4037234.91 |
| Lactate 2d (5) | 47735329 | 102 | 467993.422 |
| Lactate 20d (1) | 32781371 | 71 | 461709.451 |
| Lactate 20d (2) | 24343246 | 54 | 450800.852 |
| Lactate 20d (3) | 30196044 | 63 | 479302.285 |
| Lactate 20d (4) | 35973965 | 51 | 705371.863 |
| Lactate 20d (5) | 35221564 | 59 | 596975.661 |

| Supplementary Table S4. ROs quantification data. | | | | |
| --- | --- | --- | --- | --- |
| Sample | **Total Pixels** | **Total Pixels - background** | **Confluence %** | **Normalized Pixels/ Confluence** |
| Control (1) | 1493788 | 1341770 | 40.682 | 32981.90846 |
| Control (2) | 7227530 | 5707350 | 41.009 | 13917.31083 |
| Control (3) | 1991343 | 1839325 | 38.006 | 48395.64806 |
| Lac 2d (1) | 2104995 | 1952977 | 29.376 | 66482.06019 |
| Lac 2d (2) | 2181574 | 2029556 | 31.510 | 64409.90162 |
| Lac 2d (3) | 3385323 | 3233305 | 34.751 | 93042.07073 |
| Lac 20d (1) | 3263422 | 3111404 | 45.259 | 68746.63603 |
| Lac 20d (2) | 3888975 | 3736957 | 43.612 | 85686.43951 |
| Lac 20d (3) | 4338145 | 4186127 | 50.064 | 83615.51214 |
|  |  |  |  |  |
| Background | 1520180 |  |  |  |

| Supplementary Table S5. Cytotoxicity quantification data. | |
| --- | --- |
| Sample | **Cytotoxicity %** |
| Control (1) | 1.5896 |
| Control (2) | 1.9452 |
| Control (3) | 1.8830 |
| Control (4) | 1.8201 |
| Lactate 2d (1) | 2.1713 |
| Lactate 2d (2) | 2.2132 |
| Lactate 2d (3) | 2.1719 |
| Lactate 2d (4) | 2.1107 |
| Lactate 20d (1) | 2.2629 |
| Lactate 20d (2) | 2.4883 |
| Lactate 20d (3) | 2.5120 |
| Lactate 20d (4) | 2.3930 |

| **Supplementary Table S6.** Normality test results for experiments on Tables S2, S3, S4 and S5 | | | | |
| --- | --- | --- | --- | --- |
| **Table S2 (Shapiro-Wilk test)** | | | | |
| **Condition** | **HG** | **HG+lac** | **LG** | **LG+lac** |
| W | 0.9290 | 0.9940 | 0.8583 | 0.8870 |
| P value | 0.4850 | 0.8523 | 0.2628 | 0.3454 |
| Passed normality test (alpha=0.05)? | Yes | Yes | Yes | Yes |
| P value summary | ns | ns | ns | ns |
| **Table S3 (Shapiro-Wilk test)** | | | | |
| **Condition** | **Control** | **Lactate 2d** | **Lactate 20d** |  |
| W | 0.9089 | 0.8453 | 0.8425 |  |
| P value | 0.4763 | 0.1800 | 0.1720 |  |
| Passed normality test (alpha=0.05)? | Yes | Yes | Yes |  |
| P value summary | ns | ns | ns |  |
| **Table S4 (Shapiro-Wilk test)** | | | | |
| **Condition** | **Control** | **Lactate 2d** | **Lactate 20d** |  |
| W | 0.9963 | 0.8040 | 0.8402 |  |
| P value | 0.8834 | 0.1240 | 0.2145 |  |
| Passed normality test (alpha=0.05)? | Yes | Yes | Yes |  |
| P value summary | ns | ns | ns |  |
| **Table S5 (Shapiro-Wilk test)** | | | | |
| **Condition** | **Control** | **Lactate 2d** | **Lactate 20d** |  |
| W | 0.8985 | 0.9308 | 0.9112 |  |
| P value | 0.4238 | 0.5992 | 0.4888 |  |
| Passed normality test (alpha=0.05)? | Yes | Yes | Yes |  |
| P value summary | ns | ns | ns |  |
